# Off-resonance insensitive LGE MRI for imaging ventricular scar without image artifacts induced by cardiac devices

**DOI:** 10.1186/1532-429X-16-S1-O54

**Published:** 2014-01-16

**Authors:** Ravi Ranjan, Eugene G Kholmovski, Eun-Kee Jeong, Kyungpyo Hong, Joshua Blauer, Brent D Wilson, Christopher J McGann, Daniel Kim

**Affiliations:** 1 Division of Cardiology, Internal Medicine, University of Utah, Salt Lake City, Utah, USA; 2 UCAIR, Radiology, University of Utah, Salt Lake City, Utah, USA; 3 Department of Bioengineering, University of Utah, Salt Lake City, Utah, USA

## Background

Late gadolinium enhanced (LGE) MRI is the gold standard test for non-invasive detection of myocardial scar. Many VT ablation candidates who would derive benefit from LGE MRI do not undergo cardiac MRI largely due to image artifacts generated by cardiac devices. A recent study reported improved LGE MRI for patients with implantable cardiac devices using a custom-made wideband adiabatic inversion-recovery (IR) pulse [1]. The purpose of this study was to implement off-resonance-insensitive LGE MRI based on commercially available IR pulse for imaging ventricular scar without image artifacts induced by cardiac devices.

## Methods

We implemented cardiac-device-insensitive LGE MRI by modifying a commercially available adiabatic IR pulse (Siemens_external_RF_file:IR10240H180.IR180_36B1_2) with the following parameters:β = 750radians/s, μ = 10(dimensionless), pulse duration = 6.1 ms. We designed the IR pulse to achieve B_1_+ of 1050 Hz and 779 Hz at 1.5T and 3T, respectively, in order to achieve adiabaticity within the RF amplifier and SAR limits. Standard and wideband LGE MRI pulse sequences were evaluated in phantoms and seven canines (with ICD placed 10 cm away from the heart) with myocardial lesions created by radio-frequency ablation at 3T, as well as in one patient with ICD at 1.5T. Both LGE MRI pulse sequences used the same standard imaging parameters, except for the IR pulse. Two readers independently evaluated the image quality(1-5;worst-best) and artifact level(1-5;least-most) using a 5-point Likert scale. After administration of TTC, animals were euthanized for heart removal and gross pathology.

## Results

**Figure 1:**
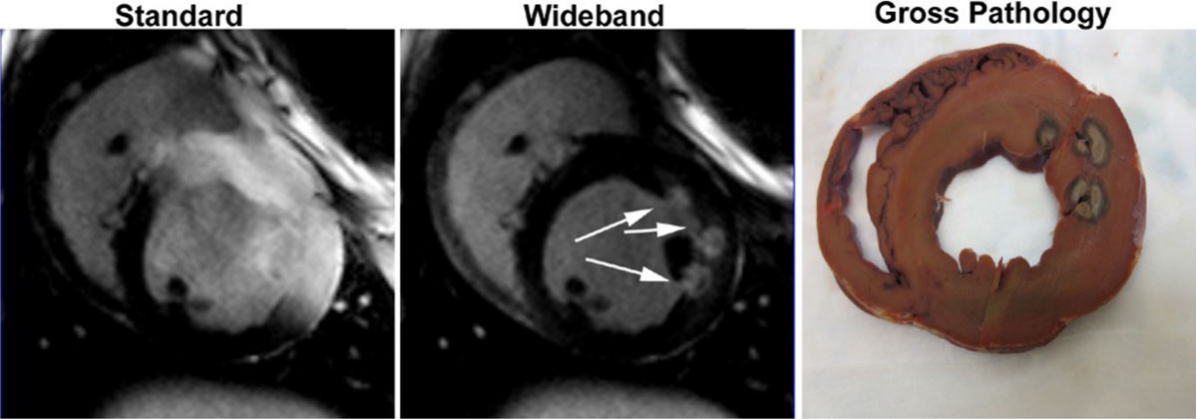
**ECG-gated segmented LGE images of a dog with RF lesions acquired with the (left) standard and (middle) wideband IR pulses at 3T**. Unlike standard LGE MRI which exhibited image artifacts induced by the device, wideband LGE MRI did not exhibit significant image artifacts and enabled complete visualization of RF lesions (arrows). Moreover, the wideband LGE image correlated well with (right) gross pathology, whereas the standard LGE image did not.

**Figure 2:**
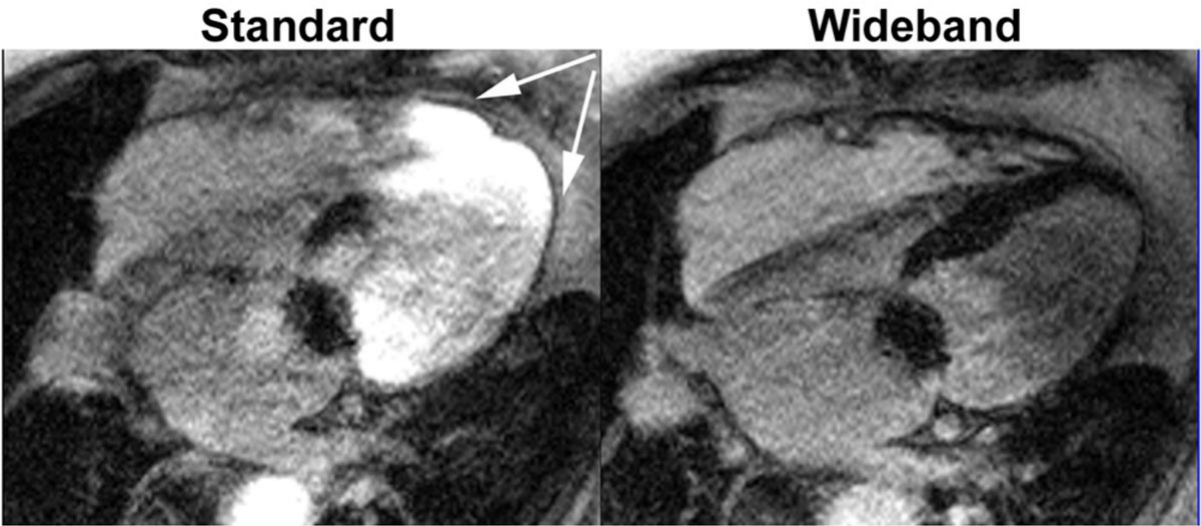
**(Left) Standard and (right) wideband LGE images of a patient with ICD acquired at 1**.5T. Compared with standard LGE MRI which exhibited significant artifacts (arrows), wideband LGE MRI suppressed image artifacts induced by an ICD and enables visualization of hyper-enhancement in the thinned lateral wall with higher diagnostic confidence.

Compared with the standard IR pulse (with FWHM = 1 kHz), the wideband IR pulse had FWHM = 4 kHz, which is a 4-fold increase in frequency bandwidth. Compared with standard LGE MRI, wideband LGE MRI yielded no significant image artifacts (Figure 1) and agreed better with gross pathology. In 7 animals (37 LGE images with different RF lesions per plane), wideband LGE MRI yielded significantly (p < 0.001) higher image quality score (3.7 ± 0.8) than standard LGE MRI (2.1 ± 0.7), and significantly (p < 0.001) lower artifact level (2.1 ± 0.8) than standard LGE MRI (4.0 ± 0.6). In one patient with ICD at 1.5T, standard LGE MRI exhibited significant image artifacts, whereas wideband LGE MRI did not (Figure 2). In human subjects, standard LGE MRI yielded total SAR of 15% and 46% at 1.5T and 3T, respectively, whereas wideband LGE MRI yielded total SAR of 20% and 31% at 1.5T and 3T, respectively.

## Conclusions

Wideband LGE MRI suppressed image artifacts induced by ICD and enabled complete visualization of myocardial scars in canines and humans within the RF amplifier and SAR limits.

## Funding

Ben B. and Iris M. Margolis Foundation.

## References

[B1] Rashid S Radiology in press

